# Non-malignant occupational respiratory diseases and climate change

**DOI:** 10.5588/ijtld.23.0131

**Published:** 2023-11-01

**Authors:** M. C. D’Ovidio, A. Lancia, P. Melis, N. Vonesch, P. Tomao, C. Grandi, I. Annesi-Maesano

**Affiliations:** 1 Department of Occupational and Environmental Medicine, Epidemiology and Hygiene, Italian Workers’ Compensation Authority, Monte Porzio Catone, Rome; 2 Department of Environmental Biology, Sapienza University of Rome, Rome, Italy; 3 Institut Desbrest of Epidemiology and Public Health, University of Montpellier and Institut national de la santé et de la recherche médicale, Department of Allergic and Respiratory Disease, Montpellier University Hospital, Montpellier, France

**Keywords:** global warming, work-related respiratory diseases, outdoor working, air pollution, indoor settings

## Abstract

**BACKGROUND:**

Respiratory diseases of infectious, allergic, neoplastic or degenerative origin are due to the interaction of environmental and occupational risk factors, individual susceptibility and other co-factors and comorbidities. Asthma and other respiratory pathologies can be worsened by climate change and exposure to other agents in occupational environments.

**METHODS:**

PubMed and Scopus, and several websites on public and occupational health were queried to find publications and documents on work-related respiratory diseases, asthma, rhinitis, chronic obstructive pulmonary disease (COPD), pneumoconiosis and allergic alveolitis in association with climate change.

**RESULTS:**

Most of the retrieved articles concerned asthma (75 in Scopus), while the other topics were less frequently covered in the scientific literature, with a maximum of 29 papers for rhinitis and 23 for COPD. The most important terms highlighted by the word clouds were ‘health’, ‘air’, ‘pollution’, and, only for asthma and rhinitis, ‘pollen’ and ‘allergic/allergy’. Website data on public and occupational health, and climate change were reported.

**CONCLUSIONS:**

Assessment and management of respiratory diseases that recognise occupational exposures should be improved, and more research into integrated approaches should be favoured. Health surveillance practices for workers exposed to agents that cause respiratory diseases should be implemented. The development of biomarkers of exposure, effect and susceptibility needs further study.

## Abstract (fr)

**CONTEXTE:**

Les maladies respiratoires d’origine infectieuse, allergique, néoplasique ou dégénérative sont dues à l’interaction de facteurs de risque environnementaux et professionnels, à la susceptibilité individuelle et à d’autres cofacteurs et comorbidités. L’asthme et d’autres pathologies respiratoires peuvent être aggravés par le changement climatique et l’exposition à d’autres agents dans les environnements professionnels.

**MÉTHODES:**

PubMed et Scopus, ainsi que plusieurs sites web sur la santé publique et professionnelle ont été interrogés pour trouver des publications et des documents sur les maladies respiratoires liées au travail, l’asthme, la rhinite, la bronchopneumopathie chronique obstructive (BPCO), la pneumoconiose et l’alvéolite allergique en association avec le changement climatique.

**RÉSULTATS:**

La plupart des articles retrouvés concernaient l’asthme (75 dans Scopus), tandis que les autres sujets étaient moins fréquemment abordés dans la littérature scientifique, avec un maximum de 29 articles pour la rhinite et 23 pour la BPCO. Les termes les plus importants mis en évidence par les nuages de mots sont « santé », « air », « pollution » et, uniquement pour l’asthme et la rhinite, « pollen » et « allergique/allergie ». Des données de sites web sur la santé publique, la santé au travail et le changement climatique ont été rapportées.

**CONCLUSIONS:**

Il convient d’améliorer l’évaluation et la prise en charge des maladies respiratoires qui reconnaissent les expositions professionnelles, et de favoriser la recherche d’approches intégrées. Des pratiques de surveillance de la santé des travailleurs exposés à des agents responsables de maladies respiratoires devraient être mises en œuvre. Le développement de biomarqueurs d’exposition, d’effet et de susceptibilité doit faire l’objet d’études plus approfondies.

Climate change affects human health both directly and indirectly. In the case of occupational respiratory diseases, direct health effects are those attributable to a severe thermal environment (an example is given by prolonged heatwaves) or to extreme weather events (thunderstorms, floods, tornado, etc.), whereas indirect effects include those mediated by natural or human systems, such as certain vector-borne infectious diseases, diarrhoeal disease linked to water supply and safety or by changes in air quality and altered patterns of biocontaminants (allergens, moulds, etc.).^1^–^9^ Climate change affects air pollution and pollen, increasing the level of some air pollutants, extending the pollen season and increasing the level of exposure to pollen and moulds. Climate change may stimulate or aggravate respiratory diseases or enhance exposure to risk factors for respiratory diseases.^10^,^11^ Individuals with pre-existing cardiopulmonary diseases are at higher risk of suffering from climate change.

This paper offers an overview of work-related respiratory diseases in relation to climate change based on a literature search, with the aim of providing information useful for improving the management of health risks due to exposure to allergens and air pollution, suggesting new methodologic approaches and future lines of research.

## METHODS

PubMed and Scopus and a list of websites on occupational health were queried in order to obtain information on work-related respiratory diseases in relation to climate change. PubMed and Scopus literature data banks were used to conduct searches using specific free terms related to asthma, rhinitis, chronic obstructive pulmonary disease (COPD), pneumoconiosis and allergic alveolitis as defined in the literature^11^,^12^ in relation with climate change. The retrieved documents were used to generate word clouds representing the most common words. A sequential exploration across multiple websites focusing on public and occupational health, as well as climate change, was conducted. The objective was to collect information about respiratory diseases and various aspects of climate change. The websites consulted included reputable sources such as the World Health Organization (WHO), Centers for Disease Control and Prevention (CDC), National Institute for Occupational Safety and Health (NIOSH), Health and Safety Executive (HSE), Intergovernmental Panel on Climate Change (IPCC) and the United Nations Framework Convention on Climate Change (UNFCCC) platforms. The searches were conducted applying a time frame between 2001 and 2020 to explore the publications in the last 20 years. All data were collected up to 17 February 2021.

### Literature databases

In Scopus and PubMed, the search was performed looking for inserted terms in the title, abstract and keywords of papers. To achieve this, the [TITLE-ABS-KEY] search was used in Scopus and the [Title/Abstract] search in PubMed for publications dealing with the topic of climate change in relation to occupational environments and respiratory diseases. Search inputs were ‘climate change’ AND (occupational OR work*) AND asthma for the ‘Asthma’ search, ‘climate change’ AND (occupational OR work*) AND rhinitis for the ‘Rhinitis’ search, ‘climate change’ AND (occupational OR work*) AND (‘obstructive lung disease*’ OR ‘obstructive pulmonary disease*’ OR copd OR ‘chronic obstructive pulmonary disease*’) for the ‘COPD’ search, ‘climate change’ AND (occupational OR work*) AND pneumoconiosis for the ‘Pneumoconiosis’ search, ‘climate change’ AND (occupational OR work*) AND (‘allergic alveolitis’ OR ‘extrinsic alveolitis’ OR ‘hypersensitivity pneumonitis’) for the ‘Allergic Alveolitis’ search. The results were first divided in years of publication to evaluate the change in the number of published papers over the years, in terms of every year of publication. The data obtained with the search were exported in the form of text files containing titles, abstracts and keywords (if available) of the retrieved papers. The text files were prepared for word analysis by applying the following steps: extraneous details about the papers (such as author names and editorial data) and common words were removed; plural words that were considered relevant for the study were changed into singular form; all upper case letters were turned into lower case letters; relevant multi-word terms were modified to be treated as single words in the analysis by substituting spaces between words with underscores. The prepared texts were analysed using an online text analyser (https://www.online-utility.org/text/analyzer.jsp) to obtain a count of the most represented words. The same text files were used to generate word clouds using the Pro Word Cloud extension for Microsoft Word (Microsoft, Redmond, WA, USA). A different word cloud was generated for each research in each database, and the 100 most common terms were included in the clouds.

### Websites on public and occupational health and climate change

Several national and international websites on public and occupational health focusing on respiratory diseases were investigated. Due to variations in the structure of each website’s homepage and the organization of topics—either listed alphabetically or grouped according to the affected organ or apparatus in the context of respiratory diseases—the search approach had to be tailored individually for each website. The investigation was performed using free terms such as ‘asthma’, ‘rhinitis’, ‘COPD’, ‘pneumoconiosis’ and ‘allergic alveolitis diseases’. In order to target climate change matters, the search was directed towards institutions and associations that specialise in environmental and climatic affairs. The websites that were referred to for the purpose of acquiring recent information about the intersection of public and occupational respiratory health with climate change included those of the WHO, CDC, NIOSH, HSE, IPCC and UNFCCC.

### Ethics approval

This study did not require an ethical approval or informed consent as the research did not involve human subjects.

## RESULTS

### Literature databases

**Figure 1:**
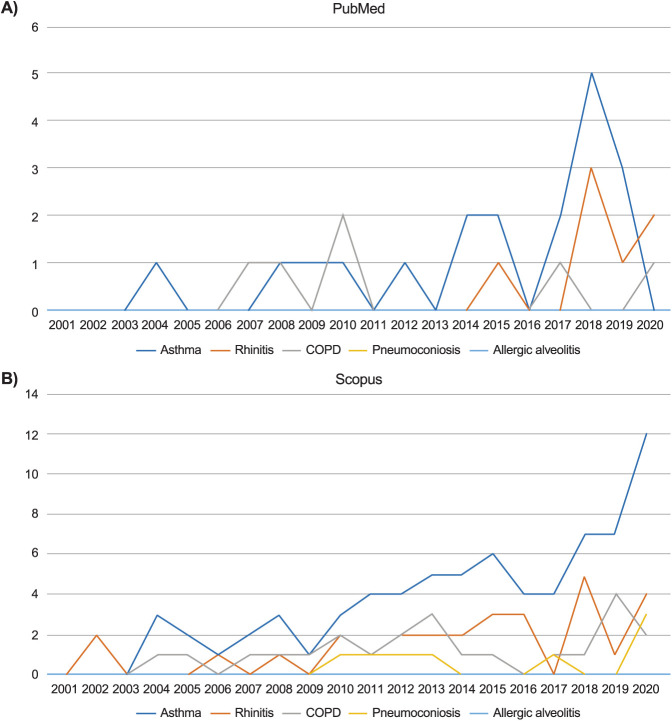
Number of published articles for every year for the searches on respiratory diseases in **A)** PubMed, and **B)** Scopus.

The search topic with the highest number of retrieved papers (Supplementary Table S1) was ‘asthma’, with 75 articles from Scopus and 19 from PubMed. Other search topics yielded fewer numbers of articles, with a similar number of documents for ‘rhinitis’ (Scopus: 29; PubMed: 9) and ‘COPD’ (Scopus: 23; PubMed: 4), while the number for ‘pneumoconiosis’ was much lower (Scopus: 8; PubMed: 0). No articles were retrieved under ‘allergic alveolitis’ from any of the databases. In terms of the annual number of publications, there was an increase in publications for ‘asthma’ in 2017 and 2018 in PubMed (Figure 1A), followed by a sudden decrease, with 0 articles in 2020. A similar but more restrained trend was observed for ‘rhinitis’, with an increase of articles in 2017 and 2018 and a subsequent decrease, but with another slight increase in 2020. There was no clear pattern for ‘COPD’, as the number of articles was very low. There was an upward trend publications on ‘asthma’ in Scopus (Figure 1B), starting in 2010, with the highest number of publications reached in 2020. Other searches did not show clearly recognisable trends, but in general, the number of articles was higher after 2010.

### Asthma

**Figure 2:**
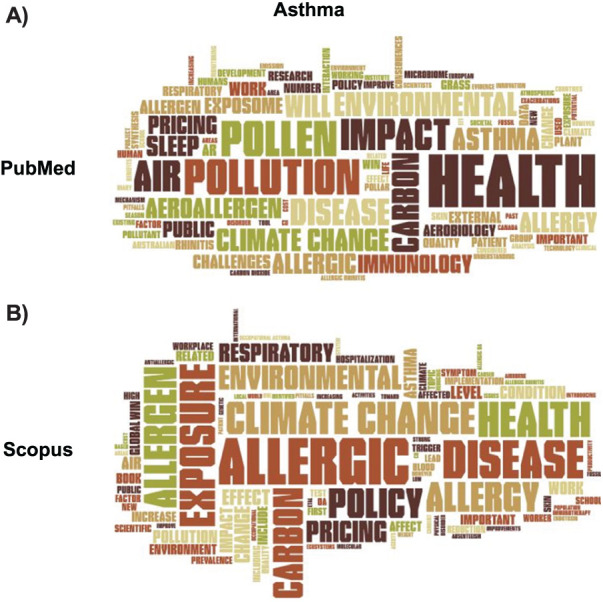
Word clouds for the ‘asthma’ search in PubMed and Scopus.

In the PubMed word cloud (Figure 2A), ‘health’ was the most prominent word, with other important terms being ‘air’, ‘climate change’, ‘pollen’, ‘disease’, ‘pollution’, ‘allergic’, ‘asthma’, ‘environmental’, ‘aeroallergen’. These results show that most of the studies relate asthma to airborne allergens, such as pollen, whose distribution is supposedly linked to climate change, but also to air pollution. There also appeared to be a focus on environmental factors linked to such themes. The Scopus word cloud (Figure 2B) similarly highlighted ‘health’ as a significantly pertinent term. Comparable essential terms to those seen in the PubMed cloud emerged as significant, such as ‘disease’, ‘climate change’, ‘air’, ‘asthma’, ‘environmental’, ‘pollution’, ‘pollen’ and ‘allergic’. Nonetheless, distinctions surfaced in the prominence of the term ‘exposure’, which held a more pronounced presence in Scopus. Furthermore, certain terms exhibited varying degrees of prevalence in PubMed compared to other terms, without necessarily being tied to the absolute frequency of their occurrence—examples include ‘air’ or ‘pollen’. A list of the 25 most frequent terms found in titles, keywords and abstract of the retrieved articles in PubMed and Scopus is given in Supplementary Table S2.

### Rhinitis

**Figure 3:**
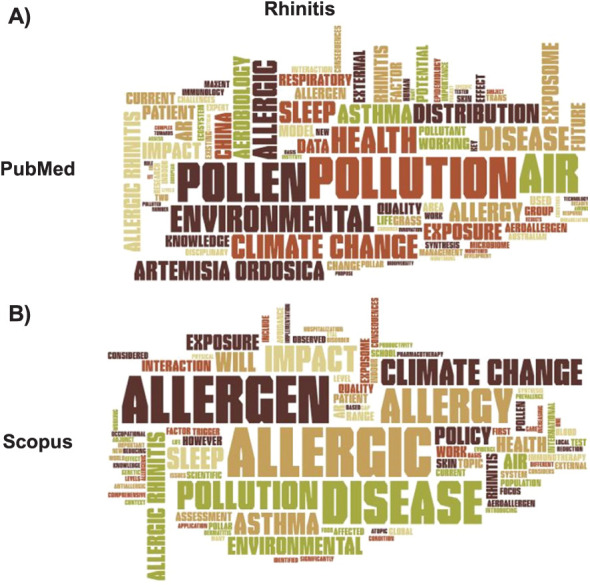
Word clouds for the ‘rhinitis’ search in PubMed and Scopus.

In the PubMed cloud (Figure 3A), the most important word was ‘air’, followed by ‘pollution’, ‘allergic’, ‘pollen’ and ‘climate change’. Among the important terms, we also found ‘allergic rhinitis’ and its acronym ‘AR’, but the presence of ‘NAR’ (non-allergic rhinitis) was also noted. In Scopus (Figure 3B) the term ‘air’ was relevant, but less important than other terms such as ‘pollen’, ‘disease’, ‘allergic’ or ‘climate change’. While both ‘allergic rhinitis’ and ‘AR’ were found, the term ‘NAR’ was notably absent from the most frequently used words. This absence suggests that papers centred around non-allergic rhinitis held relatively limited significance in Scopus. ‘Pollution’ was also less relevant, while ‘asthma’ was more frequent in Scopus than in PubMed. The list of the 25 most frequent terms found in titles, keywords and abstract of the retrieved articles in PubMed and Scopus is given in Supplementary Table S3.

### Chronic obstructive pulmonary disease

**Figure 4:**
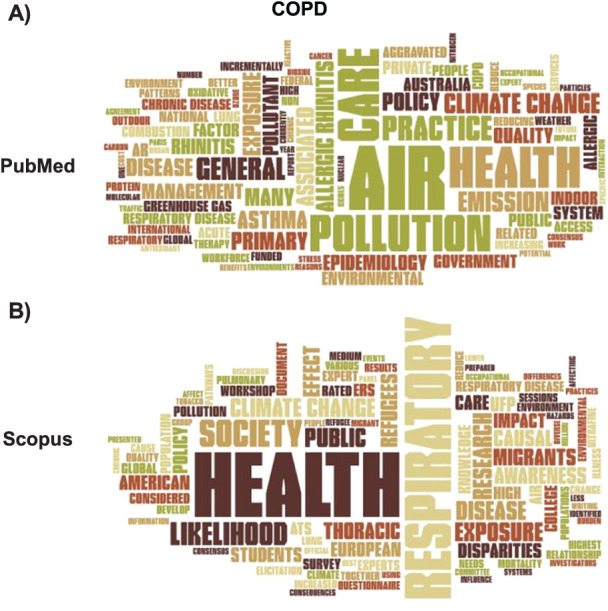
Word clouds for the ‘COPD’ search in PubMed and Scopus.

Like other word clouds based on PubMed publications, the ‘COPD’ cloud (Figure 4A) also indicated ‘air’ to be the most important word, followed by ‘health’, ‘pollution’ (another common word in all the clouds), ‘care’, ‘practice’, ‘exposure’, ‘climate change’ and ‘allergic rhinitis’. As the PubMed cloud was based on only four articles, the number of words was generally very low. In the Scopus cloud (Figure 4B), ‘health’ was by far the most relevant word, while ‘air’ was important, but not as much as in PubMed. Other important terms were ‘respiratory’, ‘climate change’, ‘disease’, ‘impact’ and ‘pollution’. The word ‘care’ was also relevant, as in PubMed. ‘Inhaler’ was also a common word. The list of the 25 most frequent terms found in titles, keywords and abstract of the retrieved articles in PubMed and Scopus is given in Supplementary Table S4.

### Pneumoconiosis

**Figure 5:**
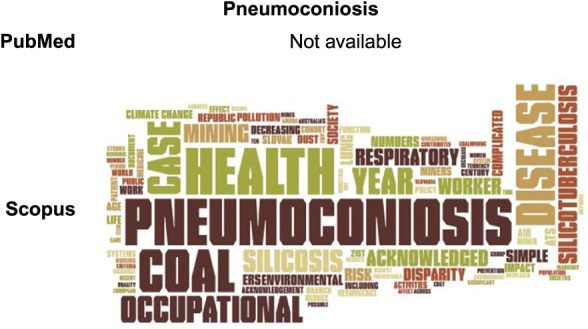
Word clouds for the ‘pneumoconiosis’ search in PubMed and Scopus.

There were no articles on pneumoconiosis retrieved in PubMed. In Scopus, the word cloud (Figure 5) was based on eight articles, with ‘health’ and ‘pneumoconiosis’ being the most relevant words. Other important words were ‘coal’ and ‘mining’, indicating that various studies focus on miners in regard to pneumoconiosis. ‘Occupational’ and ‘work’ were also well represented. There were also some specific illnesses among the most common words such as ‘silicosis’ and ‘silicotuberculosis’. However, no relation to climate change existed for pneumoconiosis.

The list of the 25 most frequent terms found in titles, keywords and abstracts of the retrieved articles in Scopus is given in Supplementary Table S5.

### Websites

Public health, occupational health and climate change were the main subject areas in the websites consulted (Supplementary Table S6). The CDC refers to ‘work-related respiratory diseases’, while the HSE refers to ‘occupational lung diseases’. Supplementary Table S7 gives the links between pneumoconiosis, asthma, COPD, lung cancer and other work-related respiratory conditions (source: CDC). Supplementary Table S7 shows the links between work-related asthma, COPD, work-related lung cancer, pneumoconiosis, silicosis, asbestos-related disease and extrinsic allergic alveolitis. The HSE gives the industry respiratory health links in alphabetical order from A to Z and the CDC reported to the National Occupational Research Agenda (NORA) industrial sectors and work-related respiratory diseases (Supplementary Table S8).

## DISCUSSION

The climate change-related burden of occupational respiratory diseases is poorly documented in literature. Occupational respiratory diseases have been directly related to climatic factors or to air pollution, pollen and moulds that are increasing due to climate change. Notifications of work-related diseases to a central health authority regarding the direct and indirect effects of climate change are also under-reported.

Several of the retrieved papers and books addressed the issue of respiratory health effects linked to climate change. A study conducted in Arizona showed that more than 390,000 adults (12%) were affected by asthma, and about 59% of adults with asthma reported attacks in recent years.^13^ According to the WHO, about 262 million people were affected by asthma in 2019,^14^ while occupational exposures contribute approximately 17% to all adult-onset asthma cases today.^15^ Based on these results, physicians in charge of allergies should take into account the increase of pollen concentrations and the longer pollen seasons.^16^ Allergies are non-communicable diseases that caused almost 300,000 deaths worldwide in 2013 due to asthma, food allergy and/or anaphylaxis, as reported by World Allergy Organization (Milwaukee, WI, USA).^16^ Moreover, it is estimated that about 300 million individuals are currently affected by asthma and/or allergic rhinitis, and by 2050 more than half of the world’s population will have an allergic disease.^16^

Fewer publications focused specifically on the impact of climate change on allergic rhinitis in relation to pollen and fungal spores, underlining the need for new policies of mitigation and adaptation to environmental changes.^17^,^18^ Climate change can trigger a shift in the start of the flowering of some plant species, such as birch and ragweed,^19^,^20^ a phenomenon that has an important effect on human health, considering the allergenicity of these types of pollen. The impact of air pollution is also considered, with its effects on both asthma and rhinitis.^21^ On the other hand, COPD has not been linked to pollen in literature, but rather to other air pollutants, such as those derived from combustion, ultrafine particles and to weather changes.^22^,^23^

An IPCC special report gives the impact of global warming of 1.5°C above pre-industrial levels in the context of strengthening the global response to the threat of climate change, and several other reports have been and will be released (https://www.ipcc.ch/) shortly.^24^ In order to increase workers and occupational health professionals’ awareness of the risk of acquiring these diseases, information and training are of fundamental importance. Drawing from the outcomes discussed earlier, the authors of this study put forth the subsequent research directions and intervention strategies. These proposals aim to enhance the evaluation and control of occupational respiratory diseases and their association with climate change—be it as causes, contributory elements or factors that amplify/aggravate the conditions.

- Development of integrated models to assess the impact of climate change on occupational respiratory health, with a focus on outdoor–indoor interchange dynamics. The following topics are of particular interest: spatial and temporal patterns of exposure to indoor pollutants in relation to both indoor and outdoor sources, optimisation of microclimatic parameters to protect workers with respiratory diseases and to prevent the occurrence of respiratory diseases, setting up of air exchanges in order to optimise physical (e.g., radon), chemical and biological pollutants removal, role/s of occupants;
- Development of approaches based on artificial intelligence to search, select, extract and treat relevant information from national and international databases with reference to the spatiotemporal patterns, as well as the environmental, occupational and lifestyle-related determinants of respiratory diseases, even in relation to the local dynamics of climatic variables;
- Improvement of the notification system and information flows of work-related diseases, accompanied by suitable information and training of the professionals concerned for a more detailed knowledge of the role and contribution of work-related exposures to the overall burden of disease;
- Development of health surveillance practices of workers exposed to agents causing respiratory diseases or exacerbating the clinical features of pre-existing respiratory diseases with increased importance accorded to health promotion and the improvement of individual resilience (for example, by providing targeted information and training on healthy lifestyles);
- Development of biomarkers of exposure, effect and susceptibility. ‘Omics’ approaches have to be electively explored in this regard for use in occupational medicine.

Overall, assessment and management of respiratory diseases that recognise occupational exposures and/or ongoing climate change variables as causes, concomitant causes or exacerbation/worsening factors should be improved. Integrated models to assess the impact of climate changes on respiratory health, with a focus on the outdoor–indoor interchange dynamics should be promoted. Health surveillance practices for workers exposed to agents that cause respiratory diseases or exacerbate the clinical features of pre-existing respiratory diseases should be implemented. The development of biomarkers for exposure, effects and susceptibility needs further study.
