# Supplementary material for: Non-malignant occupational respiratory diseases and climate change
Source: Int J Tuberc Lung Dis. 2023 Nov 1;27(11):858–63. doi: 10.5588/ijtld.23.0131 (PMC10599416; doi:10.5588/ijtld.23.0131)
Supplement: Supplementary file 1 [file iutld_ijtld_23.0131_supplementarydata1.pdf]

http://dx.doi.org/10.5588/ijtld.23.0131

SUPPLEMENTARY DATA

Non-malignant occupational respiratory diseases and climate change

M. C. D'Ovidio, [1] A. Lancia, [1,2] P. Melis, [1] N. Vonesch, [1] P. Tomao, [1] C. Grandi, [1] I. Annesi-Maesano [3]

[1] *Department of Occupational and Environmental Medicine, Epidemiology and Hygiene, Italian Workers' Compensation Authority (INAIL), Monte Porzio Catone, Rome, Italy*

[2] *Department of Environmental Biology, Sapienza University of Rome, Rome, Italy*

[3] *Institut Desbrest of Epidemiology and Public Health, University of Montpellier and Institut national de la santé et de la recherche médicale, Department of Allergic and Respiratory Disease, Montpellier University Hospital, Montpellier, France*

Supplementary Table S1 Total number of articles obtained in the two databases.

Search	Input	PubMed	Scopus
Asthma	*"climate change"* AND *(occupational OR work*)* AND *asthma*	19	75
Rhinitis	*"climate change"* AND *(occupational OR work*)* AND *rhinitis*	9	29
COPD	*"climate change"* AND *(occupational OR work*)* AND *("obstructive lung disease*"* OR "*obstructive pulmonary disease*"* OR *copd* OR "*chronic obstructive pulmonary disease*")*	4	23
Pneumoconiosis	*"climate change"* AND *(occupational* OR *work*)* AND *pneumoconiosis*	0	8
Allergic alveolitis	*"climate change"* AND *(occupational OR work*)* AND *("allergic alveolitis"* OR "*extrinsic alveolitis"* OR "*hypersensitivity pneumonitis")*	0	0

Supplementary Table S2 “Asthma” search: List of the 25 most frequent terms found in titles, keywords and abstract of the retrieved articles in PubMed and Scopus.

Asthma					
PubMed			Scopus		
	Term	Frequency		Term	Frequency
1	Health	100	1	Health	228
2	climate change	53	2	disease	147
3	air	52	3	climate change	141
4	pollen	50	4	asthma	115
5	disease	49	5	air	105
6	pollution	43	6	respiratory	99
7	asthma	42	7	environmental	98
8	effect	35	8	impact	94
9	impact	31	9	pollution	86
10	exposure	31	10	exposure	82
11	respiratory	30	11	pollen	74
12	environmental	29	12	allergic	68
13	immunology	28	13	allergen	61
14	allergic	27	14	change	60
15	allergen	23	15	climate	55
16	emission	23	16	effect	53
17	policy	22	17	allergy	50
18	public	22	18	public	48
19	allergy	22	19	energy	45
20	care	21	20	work	42
21	change	20	21	research	41
22	climate	19	22	human	41
23	work	17	23	policy	38
24	aeroallergen	14	24	global	37
25	greenhouse gas	14	25	aeroallergen	36

Supplementary Table S3 “Rhinitis” search: List of the 25 most frequent terms found in title, keywords and abstract of the retrieved articles in PubMed and Scopus.

Rhinitis					
PubMed			Scopus		
	Term	Frequency		Term	Frequency
1	air	47	1	pollen	51
2	pollution	39	2	disease	51
3	allergic	25	3	ar	50
4	pollen	25	4	allergic	48
5	climate change	23	5	climate change	45
6	ar	22	6	air	44
7	health	22	7	allergen	43
8	disease	20	8	allergic rhinitis	40
9	patient	18	9	health	40
10	factor	18	10	pollution	39
11	allergic rhinitis	18	11	asthma	36
12	allergy	17	12	allergy	34
13	exposure	17	13	exposure	32
14	environmental	16	14	environmental	31
15	nar	16	15	patient	27
16	effect	16	16	impact	26
17	asthma	15	17	change	23
18	allergen	15	18	factor	22
19	rhinitis	14	19	rhinitis	19
20	distribution	12	20	trigger	19
21	indoor	12	21	management	19
22	respiratory	11	22	respiratory	16
23	pollutant	11	23	aeroallergen	16
24	impact	10	24	year	15
25	public	10	25	nar	15

Supplementary Table S4 "COPD" search: List of the 25 most frequent terms found in title, keywords and abstract of the retrieved articles in PubMed and Scopus.

COPD					
PubMed			Scopus		
	**Term**	**Frequency**		**Term**	**Frequency**
1	air	29	1	health	89
2	health	17	2	respiratory	43
3	care	17	3	climate change	41
4	pollution	16	4	disease	40
5	practice	10	5	air	34
6	general	9	6	impact	32
7	asthma	8	7	pollution	30
8	exposure	8	8	care	26
9	climate change	8	9	exposure	24
10	emission	8	10	environmental	22
11	allergic rhinitis	7	11	effect	22
12	policy	7	12	public	21
13	primary	7	13	climate	19
14	disease	7	14	inhaler	19
15	rhinitis	6	15	research	16
16	environmental	6	16	asthma	16
17	quality	6	17	risk	16
18	pollutant	6	18	change	14
19	management	6	19	global	13
20	ar	5	20	lung	13
21	government	5	21	respiratory disease	14
22	factor	5	22	policy	12
23	public	5	23	human	12
24	private	5	24	copd	12
25	lung	5	25	environment	11

Supplementary Table S5 “Pneumoconiosis” search: List of the 25 most frequent terms found in title, keywords and abstract of the retrieved articles in Scopus. No articles were found in PubMed.

	Pneumoconiosis Scopus	
	**Term**	**Frequency**
1	health	25
2	pneumoconiosis	23
3	coal	21
4	disease	19
5	case	16
6	year	13
7	occupational	13
8	silicosis	11
9	mining	9
10	silicotubercolosis	9
11	respiratory	8
12	worker	8
13	disparity	6
14	lung	6
15	risk	6
16	simple	6
17	environmental	5
18	complicated	5
19	numbers	5
20	ats	5
21	ers	5
22	impact	4
23	climate change	4
24	slovak	4
25	work	4

Supplementary Table S6 An overview on websites on public health, occupational health, and climate change.

Public healh	Web-site
Centers for Disease Control and Prevention (CDC)	https://www.cdc.gov
European Centre for Disease Prevention and Control (ECDC)	https://www.ecdc.europa.eu/en
European Respiratory Society (ERS)	www.ersnet.org
Global Alliance Against Chronic Respiratory Diseases (GARD)	https://www.who.int/respiratory/gard/en
Italian National Institute of Health (ISS)	https://www.iss.it
Italian Ministry of Health	www.salute.gov.it
National Institute of Environmental Health Sciences (NIEHS)	https://www.niehs.nih.gov
Santé et sécurité au travail (INRS)	http://www.inrs.fr
United States Environmental Protection Agency (US EPA)	https://www.epa.gov
World Allergy Organization (WAO)	https://www.worldallergy.org
World Health Organization (WHO)	http://www.who.int

Occupational health	Web-site
American College of Occupational and Environmental Medicine (ACOEM)	https://acoem.org
European Agency for Safety and Health at Work (EU-OSHA)	https://osha.europa.eu/en
European Network For Workplace Health Promotion (ENWHP)	https://www.enwhp.org
Finnish Institute of Occupational Health (FIOH)	https://www.ttl.fi/en
French Agency for Food, Environmental and Occupational Health & Safety (ANSES)	https://www.anses.fr/en
Health and Safety Authority (HSA)	https://www.hsa.ie/eng
Health and Safety Executive (HSE)	https://hse.gov.uk
International Commission on Occupational Health (ICOH)	www.icohweb.org
Institut de Recherche Robert-Sauvé en Santé et en Securité du Travail (IRSST)	http://www.irsst.qc.ca
Institute for Work & Health (IWH)	https://www.iwh.on.ca
International Labour Organization (ILO)	https://ilo.org
Italian Workers' Compensation Authority (INAIL)	https://www.inail.it
National Institute for Occupational Safety and Health (NIOSH)	https://www.cdc.gov/niosh/index.htm
Partnership for European Research in Occupational Safety and Health	https://perosh.eu/

Climate change	Web-site
Centro Euro-Mediterraneo sui Cambiamenti Climatici (CMCC)	https://www.cmcc.it/it
Climate and Clean Air Coalition (CCAC)	https://ccacoalition.org/en
Global Climate & Health Alliance (GCHA)	https://climateandhealthalliance.org/
Environment & Health Alliance (HEAL)	https://www.env-health.org/
European Climate and Health Observatory	https://climate-adapt.eea.europa.eu/observatory
Health and climate change toolkit for project managers	https://www.who.int/globalchange/resources/toolkit/en/
Intergovernmental Panel on Climate Change (IPCC)	www.ipcc.ch
International Society of Doctors for the Environment (ISDE Italia)	https://www.isde.it/
Italian Climate Network	https://www.italiaclima.org/
National Oceanic and Atmospheric Administration (NOAA)	https://www.noaa.gov
National Snow and Ice Data Center (NSIDC)	https://nsidc.org/

United Nations Framework Convention on Climate Change (UNFCCC)	https://unfccc.int/
U.S. Environmental Protection Agency (EPA)	https://www.epa.gov
World Meteorological Organization (WMO)	https://public.wmo.int/en

Supplementary Table S7 List of links on work-related respiratory diseases (source CDC: https://wwwn.cdc.gov/eWorld/Set/Work-Related_Respiratory_Diseases/88) and occupational lung diseases (source HSE: https://www.hse.gov.uk/lung-disease/index.htm.

**Work-related respiratory diseases**	
All Pneumoconioses	https://wwwn.cdc.gov/eWorld/Grouping/All_Pneumoconioses/91
Asbestosis	https://wwwn.cdc.gov/eWorld/Grouping/Asbestosis/92
Byssinosis	https://wwwn.cdc.gov/eWorld/Grouping/Byssinosis/95
Coal Workers' pneumoconiosis	https://wwwn.cdc.gov/eWorld/Grouping/Coal_Workers_Pneumoconiosis/93
Silicosis	https://wwwn.cdc.gov/eWorld/Grouping/Silicosis/94
Unspecified and other pneumoconioses	https://wwwn.cdc.gov/eWorld/Grouping/Unspecified_and_other_pneumoconioses/96
Asthma	https://wwwn.cdc.gov/eWorld/Grouping/Asthma/97
Chronic Obstructive Pulmonary Disease (COPD)	https://wwwn.cdc.gov/eWorld/Grouping/Chronic_Obstructive_Pulmonary_Disease_COPD/98
Lung cancer	https://wwwn.cdc.gov/eWorld/Grouping/Lung_cancer/103
Malignant mesothelioma	https://wwwn.cdc.gov/eWorld/Grouping/Malignant_mesothelioma/100
Pneumonia and/or influenza	https://wwwn.cdc.gov/eWorld/Grouping/Pneumonia_andor_influenza/104
Respiratory tuberculosis	https://wwwn.cdc.gov/eWorld/Grouping/Respiratory_tuberculosis/102
Hypersensitivity pneumonitis	https://wwwn.cdc.gov/eWorld/Grouping/Hypersensitivity_pneumonitis/101
Other Work-Related Respiratory Conditions	https://wwwn.cdc.gov/eWorld/Grouping/Other_Work-Related_Respiratory_Conditions/99
**Occupational lung diseases**	
Work-related asthma	https://www.hse.gov.uk/asthma/index.htm
COPD	https://www.hse.gov.uk/copd/index.htm
Work-related lung cancer	https://www.hse.gov.uk/cancer/index.htm
Pneumoconiosis	https://www.hse.gov.uk/lung-disease/pneumoconiosis.htm
Silicosis	https://www.hse.gov.uk/lung-disease/silicosis.htm
Asbestos-related disease	https://www.hse.gov.uk/asbestos/index.htm
Extrinsic allergic alveolitis	https://www.hse.gov.uk/lung-disease/extrinsic-allergic-alveolitis.htm

Supplementary Table S8 Industry respiratory health links A-Z (source: HSE https://www.hse.gov.uk/lung-disease/a-z.htm) and NORA Industrial Sectors and Work-Related Respiratory Diseases (source CDC: https://wwwn.cdc.gov/eWorld/Set/Work-Related_Respiratory_Diseases_by_NORA_Industrial_Sectors/89

**Industry respiratory health**	
Agriculture	https://www.hse.gov.uk/lung-disease/agriculture.htm
Cement and concrete manufacture	https://www.hse.gov.uk/lung-disease/cement-concrete-manufacture.htm
Construction	https://www.hse.gov.uk/lung-disease/construction.htm
Engineering	https://www.hse.gov.uk/lung-disease/engineering.htm
Electronics (Soldering)	https://www.hse.gov.uk/lung-disease/electronics-soldering.htm
Glass and glazing	https://www.hse.gov.uk/lung-disease/glass-glazing.htm
Hairdressing	https://www.hse.gov.uk/lung-disease/hairdressing.htm
Heavy clay and bricks	https://www.hse.gov.uk/lung-disease/heavy-clay-bricks.htm
Molten metals (Foundries)	https://www.hse.gov.uk/lung-disease/molten-metals-foundries.htm
Motor vehicle repair	https://www.hse.gov.uk/lung-disease/motor-vehicle-repair.htm
Plastics	https://www.hse.gov.uk/lung-disease/plastics.htm
Printing	https://www.hse.gov.uk/lung-disease/printing.htm
Quarries	https://www.hse.gov.uk/lung-disease/quarries.htm
Refractories	https://www.hse.gov.uk/lung-disease/refractories.htm
Stoneworker	https://www.hse.gov.uk/lung-disease/stonemasonry.htm
Surface engineering	https://www.hse.gov.uk/lung-disease/surface-engineering.htm
Textiles and laundries	https://www.hse.gov.uk/lung-disease/textiles-laundries.htm
Waste management and recycling	https://www.hse.gov.uk/lung-disease/waste-management-recycling.htm
Welding	https://www.hse.gov.uk/welding/index.htm
Woodworking and furniture	https://www.hse.gov.uk/lung-disease/woodworking-furniture.htm
**NORA Industrial Sectors and Work-Related Respiratory Diseases**	
Agriculture, Forestry, and Fishing Sector (AFF)	https://wwwn.cdc.gov/eWorld/Grouping/Agriculture_Forestry_and_Fishing_Sector_AFF/108
Construction (CON)	https://wwwn.cdc.gov/eWorld/Grouping/Construction_CON/109
Healthcare & Social Assistance (HAS)	https://wwwn.cdc.gov/eWorld/Grouping/Healthcare__Social_Assistance_HAS/110
Manufacturing (MNF)	https://wwwn.cdc.gov/eWorld/Grouping/Manufacturing_MNF/111
Mining (MIN)	https://wwwn.cdc.gov/eWorld/Grouping/Mining_MIN/112
Services (SRV)	https://wwwn.cdc.gov/eWorld/Grouping/Services_SRV/113
Transportation, Warehousing & Utilities (TWU)	https://wwwn.cdc.gov/eWorld/Grouping/Transportation_Warehousing__Utilities_TWU/114
Wholesale and Retail Trade (TRD)	https://wwwn.cdc.gov/eWorld/Grouping/Wholesale_and_Retail_Trade_TRD/115

Supplementary Table S9 List of jobs with the highest rates of asthma (source HSE: https://www.hse.gov.uk/asthma/trade.htm) and substances that can cause occupational asthma (source HSE: https://www.hse.gov.uk/asthma/substances.htm

**List of jobs with the highest rates of asthma**	
**Job**	**Web-site**
Baker	https://www.hse.gov.uk/asthma/bakers.htm
Vehicle spray painter	https://www.hse.gov.uk/asthma/vehicle.htm
Solderer	https://www.hse.gov.uk/asthma/solderers.htm
Woodworker	https://www.hse.gov.uk/asthma/woodworkers.htm
Healthcare worker	https://www.hse.gov.uk/asthma/healthcare.htm
Laboratory animal worker	https://www.hse.gov.uk/asthma/laboratory.htm
Agriculture worker	https://www.hse.gov.uk/asthma/agriculture.htm
Engineering worker	https://www.hse.gov.uk/asthma/engineering.htm
Welder	https://www.hse.gov.uk/asthma/welder.htm
**Substances that can cause occupational asthma**	
Alpha amylases	Azodicarbonamide
Bromelains	Carmine
Castor bean dust	Cephalosporins
Chloramine-T	Chloroplatinates and other halogenoplatinates
Chromium (VI) compounds	Cobalt (metal and compounds)
Cockroach material	Coffee bean dust
Cow epithelium/urine	Crustacean proteins
Diazonium salts	Egg proteins
Ethylenediamine	Fish proteins
Flour dust	Glutaraldehyde
Some hardwood dusts	Henna
Isocyanates	Ispaghula
Laboratory animal excreta/secreta	Latex
Maleic anhydride	Methyltetrahydrophthalic anhydride
Nickel sulphate	Opiates
Papain	Penicillins
Persulphates	Phthalic anhydride
Piperazine	Psyllium
Some reactive dyes	Rosin-based solder flux fume
Some softwood dusts	Soybean dust
Spiramycin	Storage mites
Subtilisins	Tetrachlorophthalic anhydride
Trimellitic anhydride	

Supplementary Table S10 List of occupational categories exposed to asthma (source NIOSH: https://www.cdc.gov/niosh/topics/asthma/exposures.html)

**Occupational category and examples of associated agents**											
Animal health											
Anesthetic agents	Animal proteins (from hair/fur, saliva, urine, and dander)	Biocides (gluteraldehydes and chlorhexidine)	Cleaning products	Drugs (antibiotics)	Endotoxin	Enzymes	Latex	Pollen			
Cleaning Services											
Acetic acid	Acids	Ammonia (ammonium hydroxide)	Biocides	Bleach (sodium hypochlorite)	Chloramines	Formaldehyde	Glutaraldehyde	Quaternary ammonium compounds (e.g., benzalkonium chloride)	Spray products		
Cosmetology											
Acrylic monomers	Bleaching agents	Biocides	Formaldehyde	Hair dyes	Henna	Latex	Persulfates				
Farming and Food Production											
Cereals and grains	Egg protein	Endotoxin	Enzymes	Fish and shellfish	Green coffee beans/dust	Insects	Milk protein	Plants	Plant products (natural rubber latex)	Plant proteins (grain, wheat, coffee beans/dust, tea, flours	Pollen
Healthcare											
Acrylic monomers	Aerosolized medications (e.g., pentamidine, ribavirin)	Anesthetic agents	Biocides (e.g., gluteraldehydes and chlorhexidine)	Cleaning products (e.g., quaternary ammonium compounds)	Drugs (antibiotics)	Enzymes	Latex	Metal in dental alloys	Orthopedic adhesives (methacrylates)	Psyllium	
Industrial, Manufacturing, or Construction											
Acid anhydrides (epoxy resin, dye)	Acrylic monomers (adhesives)	Aliphatic amines (e.g., ethylenediamines and ethanolamines)	Complex platinum salts	Diisocyanates (e.g., polyurethane and plastic production, spray painting, foamcoating manufacturing)	Enzymes (e.g., amlyases, lipases, proteases)	Metal dusts	Metal salts	Metalworking fluid	Western red cedar	Wood dusts or barks	
Laboratory											
Animal proteins (from hair/fur, saliva, urine, and dander)	Enzymes	Fungi	Latex								
Office and Educational											
Indoor dampness and mold (indoor environmental quality)	Vegetable gums (printer ink)										
